# Chlorophyll *a* Covalently Bonded to Organo-Modified Translucent Silica Xerogels: Optimizing Fluorescence and Maximum Loading

**DOI:** 10.3390/molecules21070961

**Published:** 2016-07-22

**Authors:** M. A. García-Sánchez, I. N. Serratos, R. Sosa, T. Tapia-Esquivel, F. González-García, F. Rojas-González, S. R. Tello-Solís, A. Y. Palacios-Enriquez, J. M. Esparza Schulz, A. Arrieta

**Affiliations:** 1 Department of Chemistry, Universidad Autónoma Metropolitana-Iztapalapa, San Rafael Atlixco 186, Col. Vicentina, Ciudad de México 09340, Mexico; natzielly@gmail.com (I.N.S.); fgg@xanum.uam.mx (F.R.-G.); srts@xanum.uam.mx (S.R.T.-S.); peay2811@hotmail.com (A.Y.P.-E.); esma@xanum.uam.mx (J.M.E.S.); 2 Department of Physics, Universidad Autónoma Metropolitana-Iztapalapa, San Rafael Atlixco 186, Col. Vicentina, Ciudad de México 09340, Mexico; rebe@xanum.uam.mx; 3 Department of Process Engineering Hydraulic, Universidad Autónoma Metropolitana-Iztapalapa, San Rafael Atlixco 186, Col. Vicentina, Ciudad de México 09340, Mexico; candy.tapi@gmail.com (T.T.-E.); fgg@xanum.uam.mx (F.G.-G.); 4 Department of Central Electron Microscopy Laboratory, Universidad Autónoma Metropolitana-Iztapalapa, San Rafael Atlixco 186, Col. Vicentina, Ciudad de México 09340, Mexico; almamireya@gmail.com

**Keywords:** chlorophyll *a*, organo-silica, sol-gel, fluorescence, hybrid substrates

## Abstract

Chlorophyll is a pyrrolic pigment with important optical properties, which is the reason it has been studied for many years. Recently, interest has been rising with respect to this molecule because of its outstanding physicochemical properties, particularly applicable to the design and development of luminescent materials, hybrid sensor systems, and photodynamic therapy devices for the treatment of cancer cells and bacteria. More recently, our research group has been finding evidence for the possibility of preserving these important properties of substrates containing chlorophyll covalently incorporated within solid pore matrices, such as SiO_2_, TiO_2_ or ZrO_2_ synthesized through the sol-gel process. In this work, we study the optical properties of silica xerogels organo-modified on their surface with *allyl* and *phenyl* groups and containing different concentrations of chlorophyll bonded to the pore walls, in order to optimize the fluorescence that these macrocyclic species displays in solution. The intention of this investigation was to determine the maximum chlorophyll *a* concentration at which this molecule can be trapped inside the pores of a given xerogel and to ascertain if this pigment remains trapped as a monomer, a dimer, or aggregate. *Allyl* and *phenyl* groups were deposited on the surface of xerogels in view of their important effects on the stability of the molecule, as well as over the fluorescence emission of chlorophyll; however, these organic groups allow the trapping of either chlorophyll *a* monomers or dimers. The determination of the above parameters allows finding the most adequate systems for subsequent in vitro or in vivo studies. The characterization of the obtained xerogels was performed through spectroscopic absorption, emission and excitation spectra. These hybrid systems can be employed as mimics of natural systems; the entrapment of chlorophyll inside pore matrices indicates that it is possible to exploit some of the most physicochemical properties of trapped chlorophyll for diverse technological applications. The data herein collected suggest the possibility of applying the developed methodology to other active, captive molecules in order to synthesize new hybrid materials with optimized properties, suitable to be applied in diverse technological fields.

## 1. Introduction

In nature, tetrapyrrole macrocycles constitute the core structure of molecules that are of vital importance in the following metallo-organic species: (i) *heme* groups, both in blood and cytochromes; (ii) iron (II) porphyrin complexes; (iii) chlorophyll, as a tetrapyrrolic macrocycle known as chlorine; and (iv) vitamin B_12_, identified as a *corrole* [1]. Furthermore, there exist a wide variety of related natural and synthetic species with similar important physicochemical, biochemical, and biological properties. Many of these macrocycles have been tested as essential parts of catalytic materials [2], sensors [3], and optical devices [4]. In fact, there is much information on the use of phthalocyanines and porphyrins complexes or their free bases that have been successfully employed as contrast agents, for the detection and elimination of bacteria, viruses, and fungi [5,6,7,8]. The application of these compounds has included the treatment of some types of cancer, especially by a methodology known as photodynamic therapy (PDT) [5,6,7]. This occurs since porphyrins, phthalocyanines, blood and chlorophyll derivatives are preferentially adsorbed and retained by bacteria, viruses or cancer cells. The most important aspect is that this kind of macrocyclic species is capable of generating, in the first place, singlet oxygen (^1^O_2_) and posteriorly radical species, when red light is irradiated over the macrocycles, to subsequently affect neighboring regions through malignant cell selective elimination [5,6,7,8]. Synthetic free or substituted tetrapyrrole macrocycles, such as the free bases of porphyrins, can manifest intense fluorescent emission in the red region of the visible spectrum that often extends into the near infrared region [9]; furthermore, it is known that radiation proceeding from this spectral region exhibits a better penetration into ill tissues [10,11].

In particular, and besides of their transcendental function during the photosynthesis process, the photophysical properties of chlorophylls have been only partially explored and selected for diverse uses in agriculture, biological science [12], solar cells [13], catalysis [14], and sensors [15], among many other technological applications [16]. Furthermore, in a similar way as the *heme* group derivatives, chlorophylls have been tested in PDT; in turn, water-soluble derivatives of chlorophyll were postulated in 1942 by Snyder [17] as potential photosensitizers for PDT. In 1984, chlorophyll derivatives (branded as *chlorins* were related to pheeophorbide *a*, i.e., a product of chlorophyll breakdown) were patented as photosensitizers for PDT [18]. Recently chlorophyll byproducts have been proven as PDT prospects with excellent results [19,20,21].

**Figure 1:**
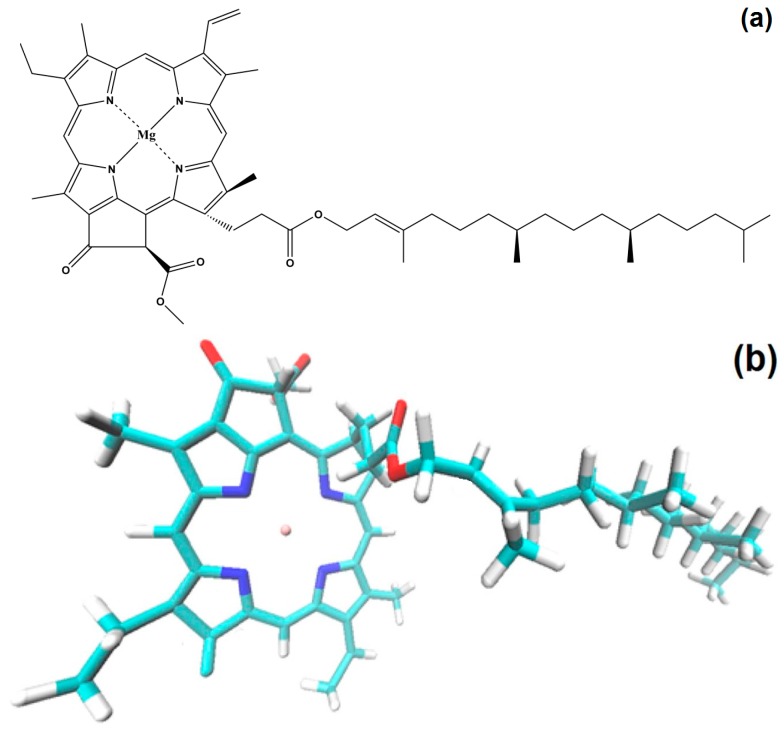
Schematic chemical structure of chlorophyll *a* (**a**) and 3D structure (**b**) visualized by employing a *Visual Molecular Dynamics 1.9.1* software program [29].

With the intention of profiting of many of the good properties displayed by tetrapyrrole macrocycles in solution, we have explored the possibility of incorporating these species inside inorganic porous matrices synthesized by the sol-gel method [22,23,24,25]. From this investigation, it was found that there exists a deleterious effect over the physicochemical properties of trapped macrocycles in porous matrices as consequence of the interactions of these species with the surface groups attached to the pore walls. With the aim of inhibiting this effect, diverse strategies have been postulated [24]. After exploring these possible options, it was found that the covalent bonding of active molecule inside organo-modified inorganic networks, synthesized by the sol-gel method, offers a convenient possibility for synthesizing hybrid systems endowed with optimized physicochemical properties [26,27]. Recently, in order to exploit and design new methods for the incorporation of natural tetrapyrrole macrocycles within solid matrices, the method developed for including synthetic macrocycles in such media was successfully extended to perform the trapping and covalent bonding of chlorophyll (Figure 1) [28] onto the pore surface of translucent monolithic organo-modified silica xerogels. These hybrid systems were synthesized using the functionalizing silicon alkoxide compound 3-aminopropyl-triethoxysilane (APTES), which was able to bound chlorophyll to the silica network synthesized from mixtures of tetraethoxysilane (TEOS) and organo-modified silicon alkoxides (OSA) through the sol-gel method. The best results were obtained when chlorophyll was bonded to the silica pore walls that were previously functionalized with *allyl* (CH_2_=CH-CH_2_-) and *phenyl* (C_6_H_5_-) groups. Based on previous evidence, in this work, we have carried out specific experiments in order to determine the maximum concentration of chlorophyll *a* that is suitable to be trapped inside monolithic silica xerogels. The objective is to find out what is the limiting mass of chlorophyll *a* that can render hybrid samples showing no aggregation between chlorophyll molecules and that, at the same time, allows an optimal fluorescence emission. Furthermore, these experiments allow determining the effect of the nature of the binding organic group over the optical response of the monolithic silica samples; the stability against aggregation of chlorophyll molecules and the displayed fluorescence of the solid hybrid networks. Among the potential practical aspects herein shown, we can mention the incorporation and exploit of the physicochemical properties of chlorophyll inserted in the pores of a xerogel structure. The methodology developed allows designing systems that have the required properties for state of the art technological applications in fields such as catalysis, compound sensing, coordination chemistry, modeling of photosynthetic systems, and performance of solar cells and medical devices.

## 2. Results and Discussion

The characterization results can be better appreciated if discussed according to two categories: (i) during the early stages of the sol-gel process, when the gellifying process is still running on; and (ii) when consolidated xerogels have been finally achieved. As described in the Experimental Section, each type of sample was prepared thrice and here we will show the most representative results concerning each specimen.

### 2.1. Phenomenology at the Start of the Sol-Gel Process

**Figure 2:**
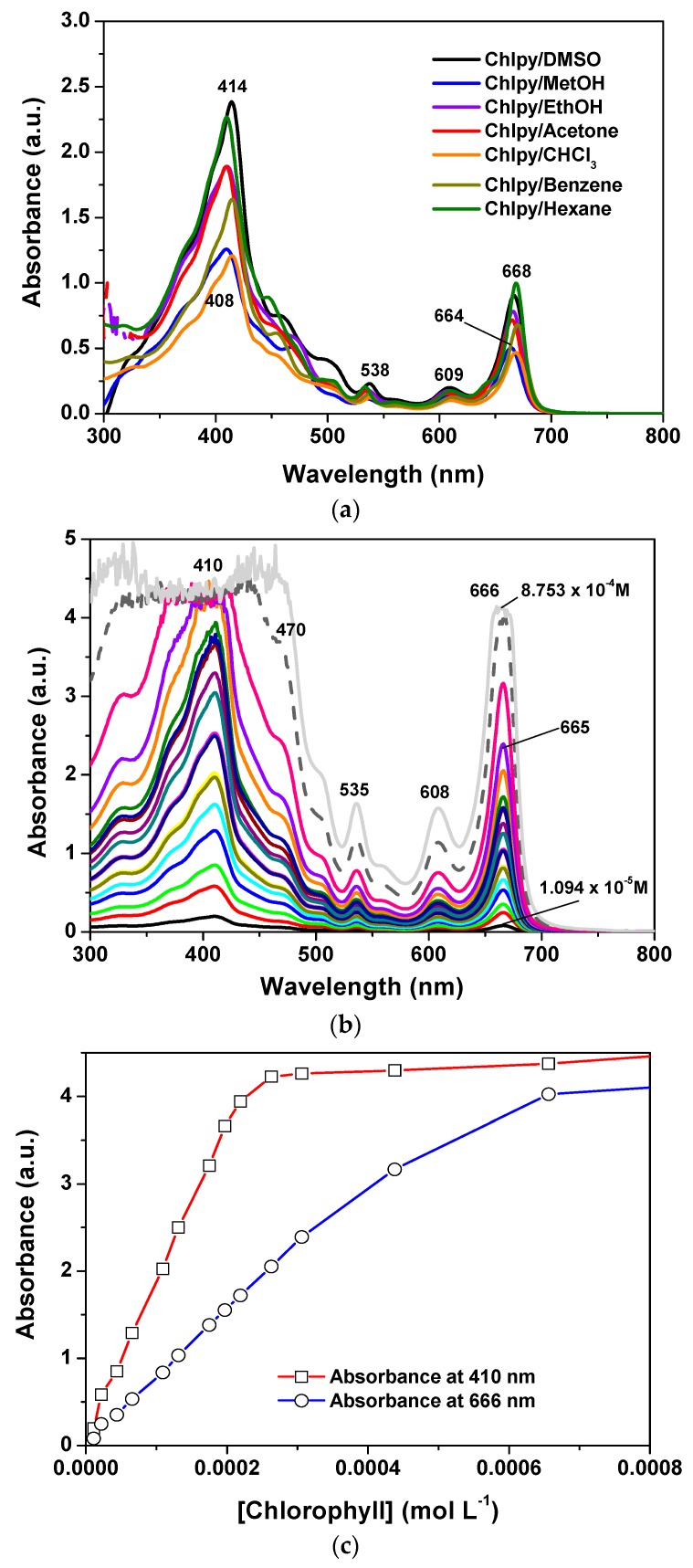
(**a**) UV-Vis absorption spectra of chlorophyll *a* solutions in solvents of different polarity; (**b**) spectra of chlorophyll at increased concentrations; and (**c**) absorbance vs. concentration plot of the signals emitted at 410 and 666 nm. Note the linearity ranges of the two signals.

The UV-Vis spectra of chlorophyll dissolved in different solvents display two prominent bands in the ranges of 408–414 nm and 664–70 nm; the first of these corresponds to the Soret band while the second corresponds to the Q_I_ band (Figure 2a,b). This pathway of signals was originally detected by Soret [30] for the *heme* group and rationalized by Guterman [31,32] for the case of porphyrins. The Soret signal has been interpreted as π-π* electronic transitions, principally due to the conjugated double bonds of the tetrapyrrole macrocycle [33,34,35].

As can be observed, the position and intensity of these bands depend on the nature of the solvent that is employed to dissolve the pigment, thus appearing slightly red shifted for the case of lowly polar solvents. The spectrum shape of chlorophyll *a* ethanolic solutions remains practically unvarying at concentrations below 5.0 × 10^−4^ M; at higher concentrations, the absorbance of the Soret and Q_I_ bands follow a linear tendency, i.e., the Lambert–Beer law is obeyed (Figure 2c). Keeping this information in mind, xerogels were synthesized in order to contain a chlorophyll concentration not exceeding the previous linearity limit.

The translucent monolithic xerogels of organo-modified silica were synthesized by a two steps procedure: in the first stage, the needed chlorophyll precursor (chlorophyll-F) was synthesized bonding this species to the 3-aminiopropyl-triethoxysilane (APTES) [26,27,28]; and, in the second stage, different amounts of the previous precursor were dissolved in ethanol and combined with water, tetraethoxysilane (TEOS), an organo substituted alkoxide (OSA = *allyl*- or *phenyl*-triethoxysilane, i.e., Ally-TEOS or Φ-TEOS) and an acidic catalyst (HCl) according to the next molar ratio sequence: 19.6:1:10^−3^:10^−3^−10^−6^ of H_2_O:TEOS + OSA:HCl:chlorophyll-F). Since the amount of chlorophyll *a* came from the addition of different volumes of the chlorophyll-F precursor to the initial mixture, each sample can be identified by the % *v*/*V_f_* used or by the X-digit identifier used in each Chl-OSA-X system. The respective initial molar concentration of chlorophyll, varied from 4.279 × 10^−5^ to 7.702 × 10^−4^ M, and is listed in Table 1 (see the Experimental Section).

The hydrolysis of ethoxy groups existing in APTES and TEOS, and the subsequent polycondensation of generated hydroxyl groups allows growing the inorganic network, which forms a sol in the first stage and a gel in the second, then becoming a solid porous matrix at the end of the sol-gel process. As previously demonstrated, no more than 1.0% *v*/*V_f_* of OSA compound has to be used in order to attain translucent silica xerogels [26,27]. At the end of the consolidation process of the gellifying mixtures in the obtained xerogels, a green coloration was evident for those samples containing a low amount of chlorophyll, while a darker green color was manifest for those specimens containing a higher amount of the pigment. In all of these samples, fractures, segregation and heterogeneities were not evident. However, as consequence of the evaporation of the liquid trapped in the porous network, and because of shrinkage, the final solid samples only represented the eleventh part of the initial liquid volume. Consequently, the concentration of chlorophyll in the final monoliths ranged from 4.707 × 10^−4^ M to 8.472 × 10^−3^ M ([chlorophyll *a*]_f_). As it was previously demonstrated, chlorophyll *a* remains bonded to the silica network through bridges proceeding from the APTES silicon alkoxide [28]; importantly, repeated washings of the resultant hybrid materials with ethanol, acetone, and chloroform showed no evidence of detached chlorophyll *a*, thus reinforcing the idea of a covalent bonding of the pigment on the pore walls.

**Figure 3:**
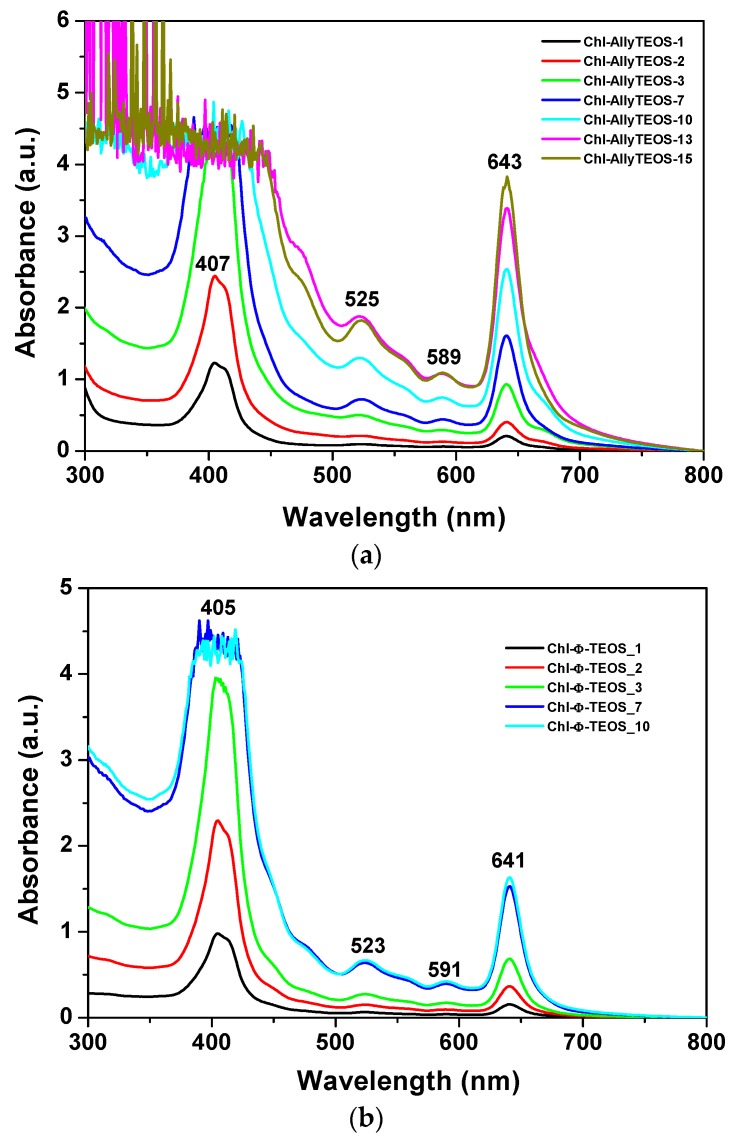
UV-Vis absorption spectra of the initial gellifying mixtures containing different concentrations of chlorophyll-APTES precursory species and 1% *v*/*V_f_* of: Ally-TEOS (**a**); and TEOS (**b**).

In the UV-Vis absorption spectra of the initial gelling mixtures that include chlorophyll functionalized with APTES (Figure 3a,b), a signal pathway can be observed. This path is similar to that associated to chlorophyll a in solution, while the associated bands appear blue shifted; the Soret signal is located at 405–407 nm and the Q_I_ emission is found at around 641–643 nm (Figure 2). In the spectra of the set of samples modified with *phenyl* groups, the Soret bands appear narrower than in the spectra of those xerogels modified with *allyl* groups. Additionally, a shallow band appears as a shoulder at the right hand side of the Q_I_ band in the *allyl* modified set of samples (Figure 3a); nevertheless, this signal is not present in the set of samples of silica modified with *phenyl* groups (Figure 3b). The existence of this signal can be associated to the dimerization of chlorophyll or to the formation of aggregates [36,37,38] in samples synthesized in the presence of the Ally-TEOS alkoxide. In other words, immersed in the gellifying sol-gel mixture, chlorophyll dimerization or aggregation occurs in the presence of *allyl* groups, but not when *phenyl* groups arise, since these last molecules inhibit the occurrence of dimerization.

The shifting of around 20 nm in the position of the Q_I_ band, and of about 5 nm for the Soret bands, could be associated to the physicochemical environment that surrounds the chlorophyll molecules in the gellifying system; additionally, this same condition starts inducing the dimerization of the pigment; however, this drastic shift cannot just be associated to the polarity of the solvent since, as shown in the spectra in Figure 2a, the polarity of the solvent can only induce a limited shift of both Q_I_ and Soret signals.

**Figure 4:**
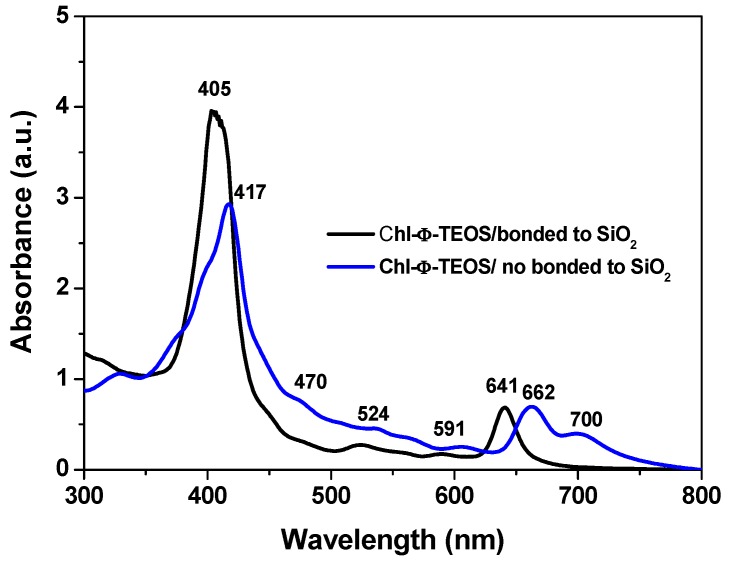
UV-Vis spectra of initial gellifying mixtures containing Φ-TEOS and 1.712 × 10^−4^ mol/L of either chlorophyll *a* or the chlorophyll-APTES precursory species.

The drastic blue shift observed in the Soret and Q_I_ bands of chlorophyll immersed in the gellifying mixtures can merely be associated to the physicochemical environment that this mixture induces over the trapped molecule. This blue shift only takes place when chlorophyll is bonded to the network through bridges proceeding from the APTES molecule previously attached to it, as shown in Figure 4. However, that shift was not observed in analogous systems where an unmodified (i.e., unfunctionalized) chlorophyll *a* solution was added to the gellifying mixture, which rendered systems in which chlorophyll was only physically trapped inside the silica pores. In this last system, the absence of molecular bridges with the growing silica network induces the aggregation of chlorophyll molecules, as suggested by the band emerging at 700 nm. Chlorophyll remains as monomeric entities up to high concentrations while dissolved in many of the most common solvents. Nonetheless, the dimerization of this pigment can be induced in some specific solvent mixtures or by using surfactants [36,38]. Then, the differences revealed by the UV-Vis of chlorophyll physically or covalently bonded to the support network suggest that the establishment of covalent unions surely affect its solvation situation and consequently the occurrence of electronic transitions; however, chlorophyll dimers or aggregates are also suitable to be trapped if needed. For these reasons, the efficiency of the method herein described can be adapted in order to trap or fix one or several chlorophyll ensembles.

### 2.2. Phenomenology at the Completion of the Sol-Gel Process

**Figure 5:**
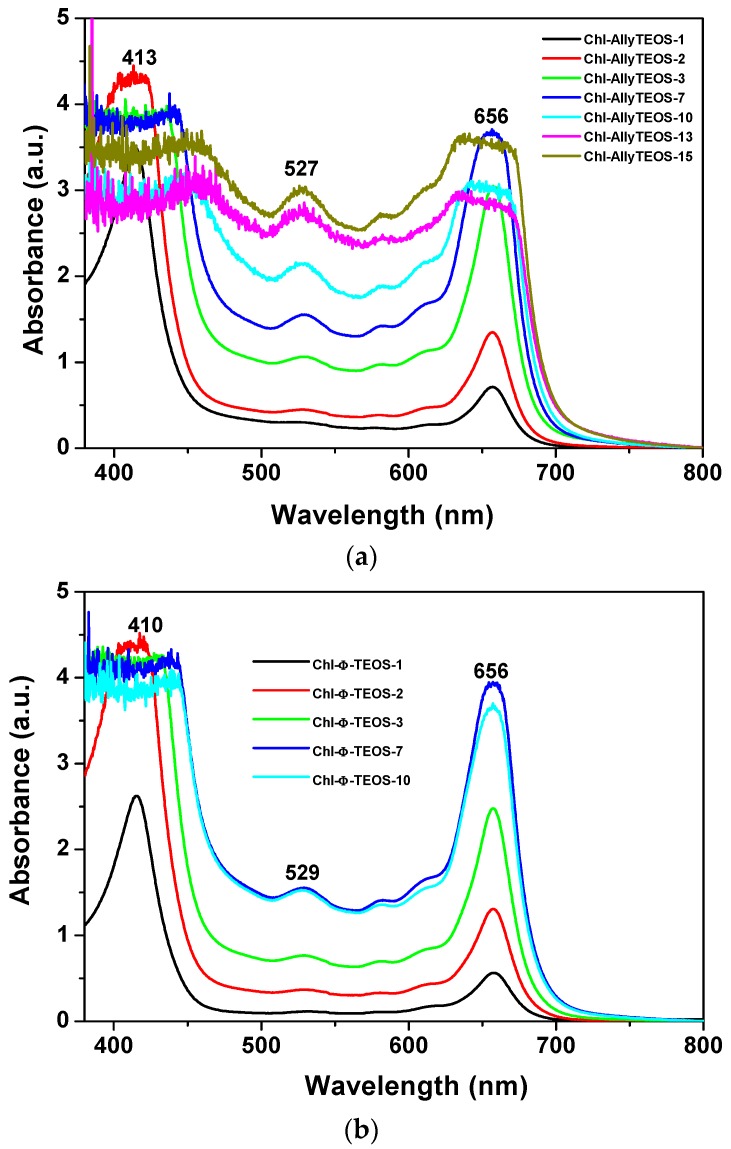
UV-Vis absorption spectra, taken at the end of the gellifying process, of chlorophyll *a* at different concentrations and covalently bonded to silica xerogels modified with: *allyl* (**a**); or *phenyl* (**b**) groups (the Chl-Ally-X or Chl-Φ-X sets).

Once the gel drying process has been completed, UV-Vis absorption measurements of chlorophyll *a* covalently bonded to silica organo substituted with *allyl* and *phenyl* groups were performed. The respective spectra sets showed the typical bands of chlorophyll somewhat widened due to gelation and xerogel formation. The Soret band appears at 414 nm, while the Q_IV_ band is located at 527 nm, and the Q_I_ band at 657 nm (Figure 5). Surprisingly, in this spectra set, the bands now appear closer to the signals observed in the UV-Vis spectrum corresponding to chlorophyll in solution (Figure 2c). However, the Soret and Q_I_ bands resulted broader at high chlorophyll concentrations, principally in the case of samples synthesized from Ally-TEOS (Figure 5a). Curiously, in this last set of samples, the bands observed at the right hand side of the Q_I_ band disappear altogether, with the exception that the Soret band resulted more intense at high chlorophyll concentrations, with a maximum intensity at around 470 nm. Additionally, a similar situation occurred with the band localized at 527 nm. Contrastingly, the Soret bands for the set of samples synthesized from Φ-TEOS (Figure 5b) experiment, a negligible distortion effect and the respective maximum intensity appears slightly red shifted for samples having a high chlorophyll contents. Clearly, the band width can be associated with the chlorophyll concentration present in each monolithic sample, which can remain trapped as monomers, dimers or aggregates. In all cases, the UV-Vis spectra of chlorophyll are not clear enough to distinguish among the previous three options. However, the changes suffered by these spectra along the sol-gel process reveal the occurrence of changes in the polarity, viscosity, and other phase properties around the macrocyclic species. In spite of these phenomena, the signal pathway and characteristic bands of the chlorophyll macrocycle resulted very similar to those observed in solution, especially at low concentrations. This situation suggests that the hydrolysis and polycondensation reactions in the gellifying mixture create fragments (or particles) that solvates, in a more or less way, the chlorophyll molecules, which gradually surround and trap them, then creating the final cavities throughout the organo-modified silica in which the pigment molecules reside. The structural situation of trapped chlorophyll molecules, together with the physical characteristics of the cavity formed at the boundaries, as well as the physicochemical conditions that are prevalent inside those pores induce an efficient or interfered display of the luminescent properties of this pigment species.

Evidently, the shape of the UV-Vis absorption spectra of the set of final xerogels modified with *phenyl* groups resulted more similar to that corresponding to the free chlorophyll species in solution than those taken before gel consolidation. At concentrations above 9.414 × 10^−4^ M (the Chl-OSA-2 final xerogels), the UV bands of the set of samples modified with *allyl* groups were broader and slightly less intense than those observed for the set of substrates modified with *phenyl* groups. This situation suggests that *allyl* groups allow for interactions that interfere with the electronic transitions of chlorophyll; in turn, the presence of *phenyl* groups around the pigment species induces a more adequate physicochemical environment, which facilitates the occurrence of the electronic transitions associated to the bonded macrocycles.

**Figure 6:**
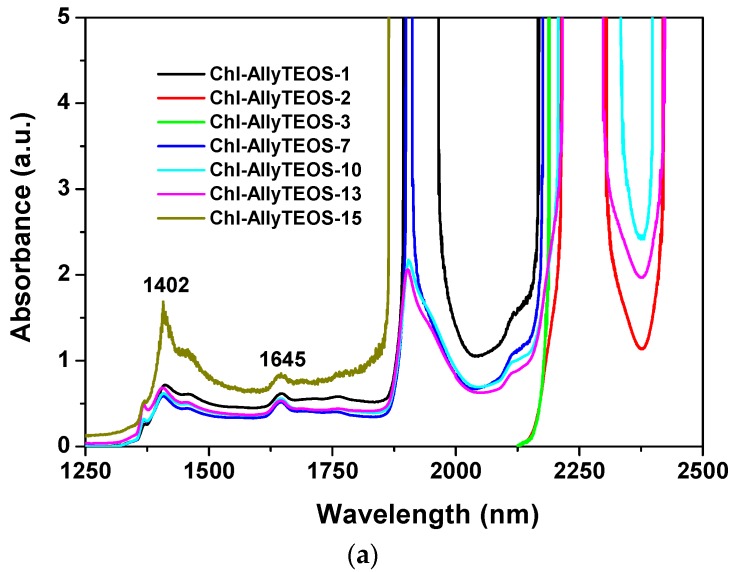

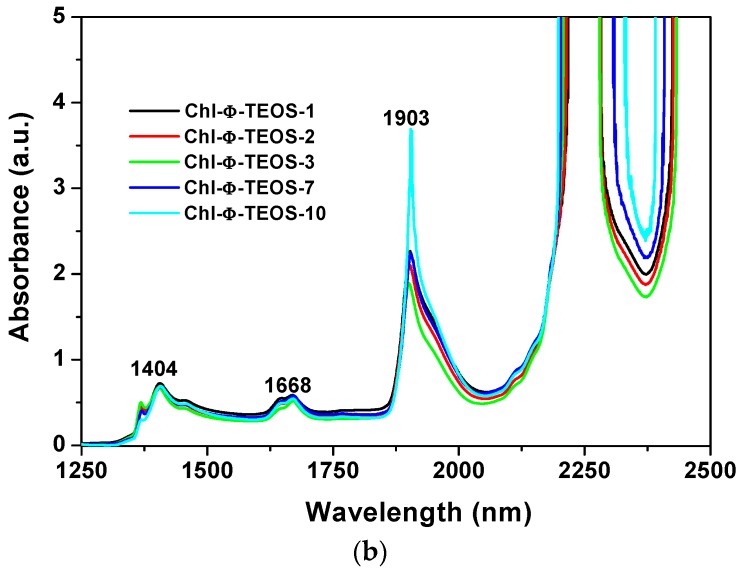
NIR absorption spectra of final xerogels including the chlorophyll covalently bonded to the pore walls of silica substrates modified with: *allyl* (**a**); or *phenyl* (**b**) groups, and at different concentrations of the chlorophyll–APTES precursor (the Chl-Ally-X or Chl-Φ-X sets). All samples were dried at 125 °C.

On the other hand, Figure 6 depicts near infrared (NIR) spectra of the sets of samples synthesized from either Ally-TEOS or Φ-TEOS, which were previously dried for one day at 125 °C. In these spectra, the band appearing at around 1900–1908 nm can be attributed to physisorbed water; in turn, the signals located at 1407–1428 nm can be associated to the first overtone (2ν_2_) of adjacent Si-OH groups and, the signal at around 2200 nm can be associated to the combined vibration of Si-OH species with the outer plane flexion of water molecules (ν_2_ + νOH (flexion)) [39]. For these reasons, this set of signals reveals the presence of Si-OH groups interacting with physisorbed water [40]. The weak peaks that should be observed between 2300 and 2400 nm, in the recently dried samples, appear masked by the intense band at 2200 nm in the rehydrated xerogels. These bands could be associated to the presence of C-H or CH2 species due to *allyl* or *phenyl* groups, as well as to the *propyl* chain of APTES or, and with a lower probability, to remnant ethoxy groups (-OC_2_H_5_) arising from partially hydrolysed silicon alkoxides [41]. However, as previously demonstrated, different signal pathways were observed from 1600 to 1800 nm due to diverse organic groups attached to the silica network after being synthesized by the sol-gel method [25,26,27]. Consequently, the signals observed in that range could be attributed to Si-C bonds of *allyl* or *phenyl* groups bonded to the silica network. Since all samples were treated in a similar way while employing the same % *v*/*V_f_* of OSA compounds, the spectroscopic intensity differences found can only be due to the chlorophyll *a* amounts and identity of organic groups that modified the silica network. In both sets of samples, the *allyl* or *phenyl* groups substituted the surface Si-OH groups and reduced their populations in the final xerogels. As was previously demonstrated, the rehydration process induces an evident NIR band pathway and intensities changes which help understanding the polar or non polar nature of the network [42]. The intensities of the peaks related to Si-OH groups interacting with physisorbed water and which appear at around 1428, 1900, and 2200 nm, suggest that, in the samples functionalized with *allyl* groups, these species have no great effect over water retention and rehydration processes, but there exists an important effect related to chlorophyll *a* concentration (Figure 6a). Apparently, in these samples *allyl* groups are not interfering the approaching of water to the polar regions of chlorophyll; for this reason, the intensity of the principal bands depends on the amount of chlorophyll attached to the matrix. However, in the NIR spectra of the other set of samples, the pathways and intensities suggest that the effect of *phenyl* groups dominates the sorption and retention processes, thus inhibiting water approaching to chlorophyll; this imposes a limit over the rehydration capacity of these samples. These results mean that the presence of *phenyl* groups determine the physicochemical environment inside of the cavities, then inducing a lower polar character or higher hydrophobicity of the network. Additionally, the NIR spectra suggest the existence of different sorption capacities of species such as water, which depend on the nature of the organic groups attached to the pore walls of the network.

**Figure 7:**
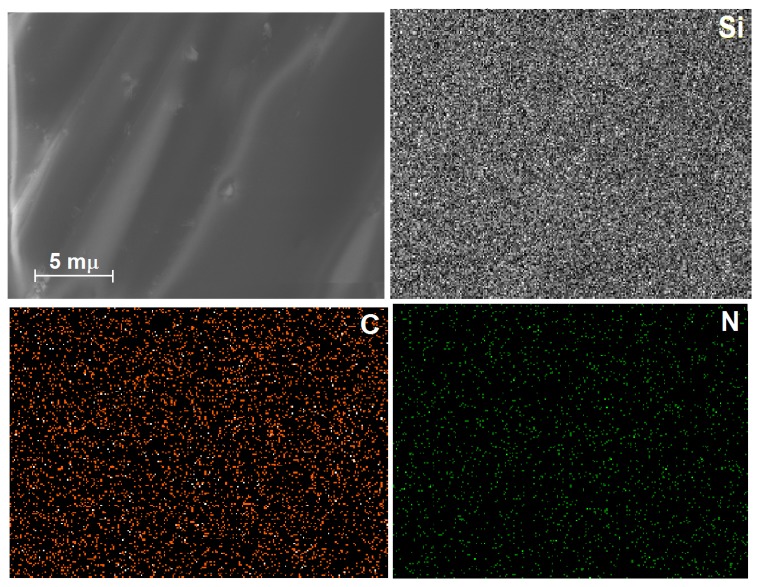
High Resolution Scanning Electron Microscopy images (HRSEM) and Dispersive X-ray Spectroscopy (EDX) mapping of silicon, carbon and nitrogen atoms in xerogels prepared from a gellifying mixture including 3 wt % of chlorophyll-APTES and 1 % *v*/*V_f_* of Ally-TEOS (Chl-Ally-3).

In the High Resolution Scanning Electron Microscopy images (HRSEM) of the xerogels synthesized with a relative low concentration of chlorophyll, while bonded to the silica walls modified via *allyl* groups (Chl-Ally-3, Figure 7), a soft surface with no obvious textural details can be observed. However, Energy-Dispersive X-Ray Spectroscopy (EDX) mapping reveals homogeneous distributions of silicon, carbon, and nitrogen. For these elements, the wt % and standard deviations were 34.6 (σ = 0.82), 9.20 (σ = 1.60), and 0.49 (σ = 0.10), respectively, and 54.57 (σ = 0.96) wt % oxygen (mapping not shown). The above weights percent of the elements, and those corresponding to the other samples, were calculated from nine spectra taken over the surface of the sample.

All these elements can be associated to the inorganic silica network in a first instance, while the carbon can be linked to the presence of organic groups attached to the substrate (i.e., *allyl* or *phenyl* groups), remnant ethoxy groups, propyl chains of the APTES molecule or to the chlorophyll skeleton. However, the uniform distribution of nitrogen observed for this sample can only be due to the four nitrogens at the center of the chlorophyll macrocycle, and to the one nitrogen of the APTES silicon alkoxide. This nitrogen represents about the 0.49 wt %, perhaps for this reason the detection of magnesium, which represents about the 2.72 wt % of the molecular weight of chlorophyll, was not easy to perform.

**Figure 8:**
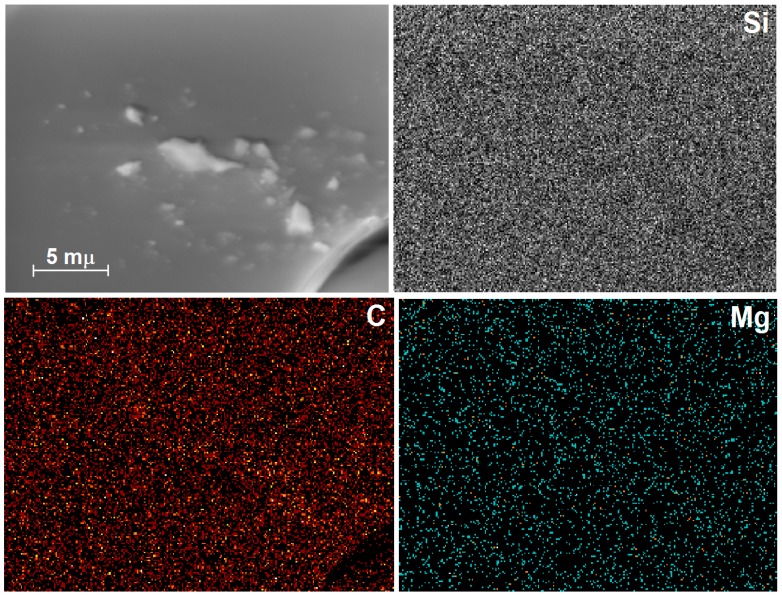
HRSEM images and EDX mapping of silicon, carbon, and magnesium atoms in a xerogel synthesized from a 1 wt % chlorophyll solution with this molecule bonded to the silica network previously functionalized with *phenyl* groups (Chl-Φ-1).

Similarly, in the HRSEM images of xerogels prepared with a low amount of chlorophyll anchored in the silica network modified with *phenyl* groups (Chl-Φ-1, Figure 8), a soft surface can be observed, without marked textural details. In the EDX mapping, a regular distribution of silicon atoms owing to the silica substrate can be observed; in turn, carbon can mainly be attributed to the organic groups attached to the silica surface and, in a lower amount, associated with the presence of the trapped chlorophyll macrocycle. Nevertheless, the most crucial result of this analysis was that, even though the very low molar concentration of chlorophyll in this sample (i.e., 4.701 × 10^−4^ M), magnesium atoms were still detected, thus demonstrating the presence of the chlorophyll *a* macrocycles in this sample. The mean wt % of C, N, O, and Si were 14.65 (std. deviation σ = 1.33), 0.80 (σ = 0.09), 52.45 (σ = 0.74, mapping not shown) and 30.98 (σ = 0.90), respectively. From these data, it was possible to calculate a 0.28 wt % of magnesium, which should be associated with the nitrogen amount determined. As was mentioned above, these elemental analyses were normalized and results are from the average of nine spectra taken over the surface of the samples.

### 2.3. Fluorescence Spectroscopy Analysis

**Figure 9:**
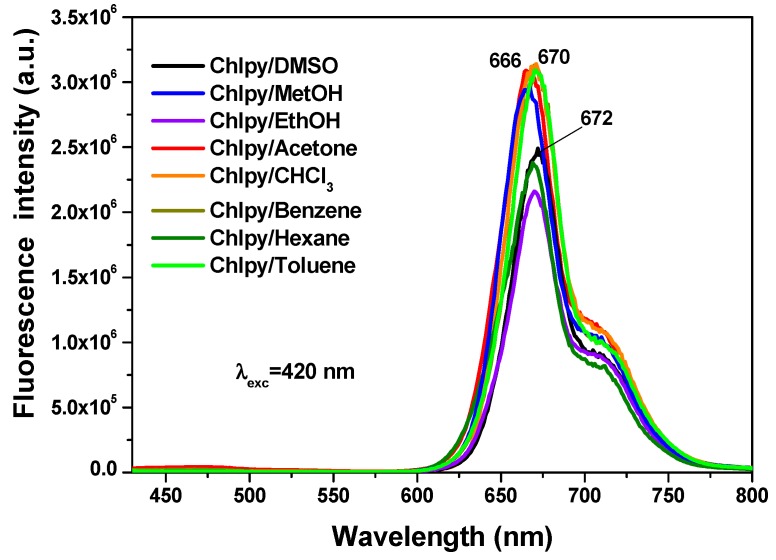
Fluorescence spectra of chlorophyll *a* in different solvents and excited with UV light of 420 nm.

In solution, the chlorophyll macrocycle displays a red fluorescence on being irradiated with ultraviolet light. For example, in different solvents, the typical chlorophyll fluorescence spectrum shows two principal bands (Figure 9) localized at around 669 nm (red region) and 707 nm (near infrared region), associated with the radiative emissions of the π-π* transitions inherent to the tetrapyrrole macrocycle of chlorin in the chlorophyll *a* structure [31,32,33,34]. In a way similar to that occurring with the absorbance spectra, the intensity and position of the emission depend on the identity of the solvent in which the macrocycle is being dissolved.

**Figure 10:**
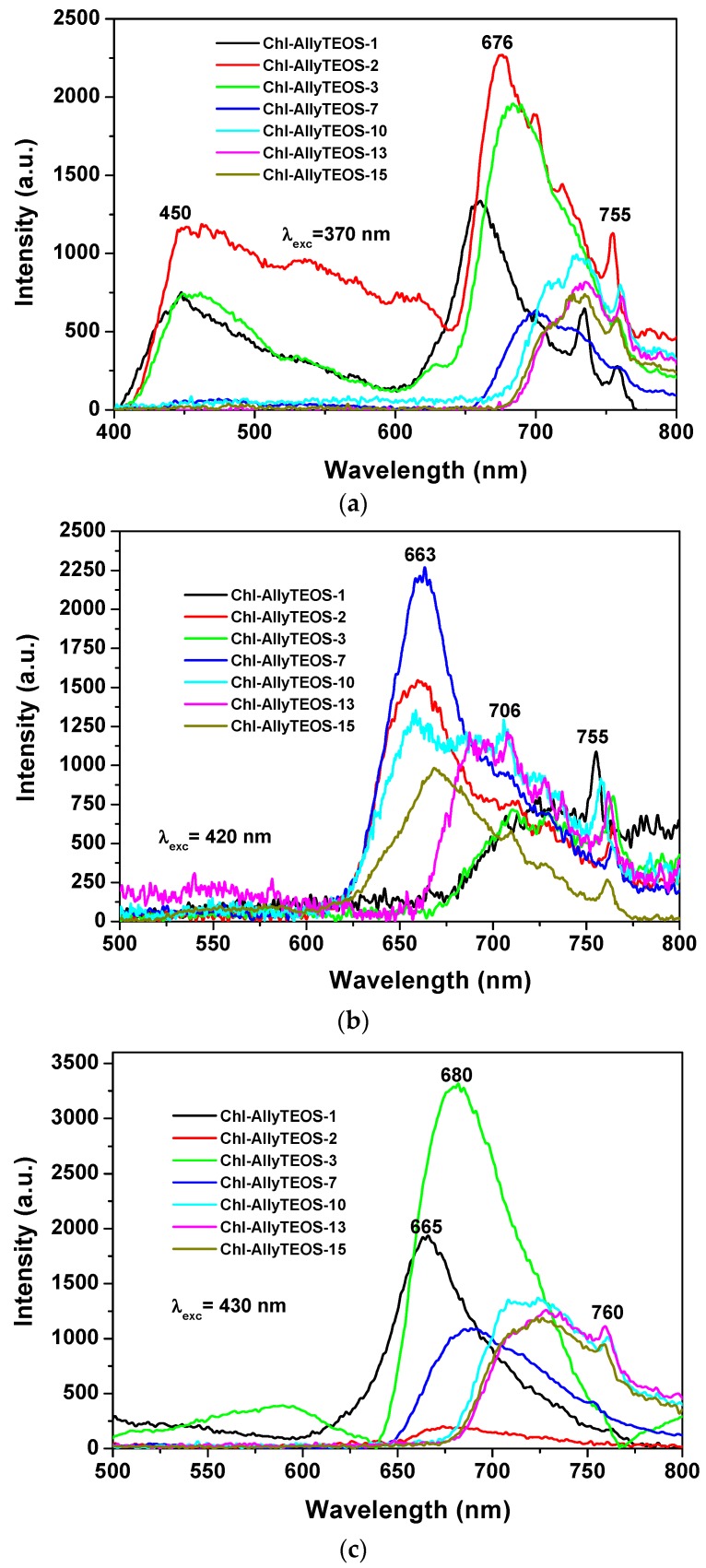
Emission and excitation spectra of chlorophyll *a* covalently bonded to the silica surface modified with *allyl* groups at different pigment loading: (**a**) emission spectra obtained with λ_exc_ = 370 nm; (**b**) emission spectra at λ_exc_ = 420 nm; and (**c**) emission spectra at λ_exc_ = 430 nm.

In the case of silica xerogels modified with *allyl* groups (Chl-Ally-X), the fluorescence spectrum was obtained through excitation with light of 370 nm (λ_ex_), there exist two emission regions between 400 and 600 nm, which are attributed to the xerogel network (Figure 10a). In turn, more intense signals from 600 nm to 800 nm can be ascribed to chlorophyll *a* molecules. In this last set of signals, the most evident peaks appear at 676 nm, 729 nm, and 755 nm. As was stated before, the emission due to the silicon oxide network can becomes the more intense (even more than that of chlorophyll) when it is excited with light of 370 nm; nevertheless, both species (SiO_2_ and chlorophyll) show significant emissions. In the same sense, the spectra shown in Figure 10b were obtained by using an excitation light of 420 nm, there it can be observed a broad band beginning at 620 nm and ending at 800 nm. A maximum intensity at 650 nm was detected, accompanied by a peak that extended to 800 nm. As expressed before, these transitions are typical of porphyrin macrocycles and are associated to the aromatic nature of the tetrapyrrole macrocycle; then, the principal signal corresponds to the Soret band (400 nm–420 nm), which is accompanied by the broad band at around 650 nm. In the excitation spectrum, two bands are observed, one at 400 nm and another one at 520 nm, the corresponding peaks are located in the absorption spectrum in the blue and green regions, respectively. When exciting samples with a 420 nm wavelength light, the fluorescence intensity results highest for the silica xerogels modified with *allyl* groups and synthesized by using more than 3 wt % of the precursory chlorophyll solution (i.e., more than 1.883 × 10^−4^ M of chlorophyll *a* in the final xerogels). A similar situation occurs when samples were excited with a 430 nm wavelength (Figure 10c); besides, a more intense and narrow band was observed between 600 and 800 nm; additionally, a bathochromic shift was detected, whose intensity diminished as the chlorophyll loading increased. In this set of samples was clear the existence of two UV spectra pathways: one with a maximum intensity at around 665 to 680 nm for samples endowed with low chlorophyll concentration and a second one in which the maximum intensity appears red shifted at around 630 nm. This phenomenon can be caused by the interaction between chlorophyll and the silica network, but more probably to dimerization or aggregation of the chlorophyll molecules. As it was previously found by diverse authors, this phenomenon is of a less intensity at the limit of the NIR region; usually centered between 730 and 750 nm and which is associated to a strong excitonic coupling between the tetrapyrrole macrocycles [43] existing in dimeric or aggregated forms [36,37,38]. In a similar way as that followed for the interpretation of the UV-Vis spectra, the next situation is related to the observation of some signals in the spectra of samples including a high amount of chlorophyll, which suggest the entrapping of one or more pigment molecule in the xerogel cavities.

**Figure 11:**
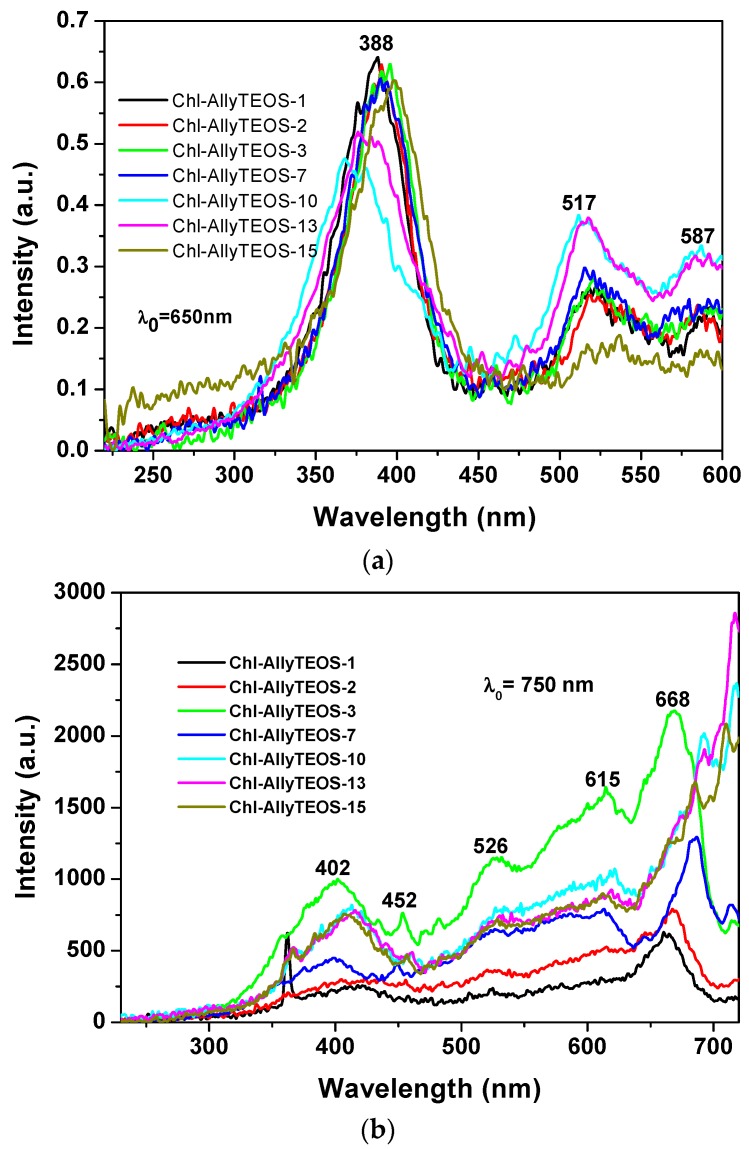
(**a**) Excitation spectra determined at λ_ex*c*_ = 650 nm; and (**b**) excitation spectra at λ_exc_ = 750 nm of the samples synthesized using Ally-TEOS alkoxide (Chl-Ally-X).

A more regular situation was observed when samples were excited with light of 650 nm (Figure 11a): in these spectra set, an intense band was observed at around 388 nm together with two other signals of lower intensities at 517 and 587 nm. The first band can be attributed to the silica network, while the other two could be associated to the Q absorbance bands of chlorophyll. However, when the SiO_*2*_ samples modified with *allyl* groups (Ally-TEOS) were excited with a 750 nm wavelength light, a set of bands of increasing intensity was observed in the region extending from 400 to 615 nm. These signals can be associated, again, to their counterpart in the absorbance spectrum (i.e., a shallow Soret band and typical Q bands), especially the band of relatively low intensity, and appearing at around 668 nm, can be associated to chlorophyll, which experiments a red shift as the chlorophyll concentration starts increasing.

**Figure 12:**
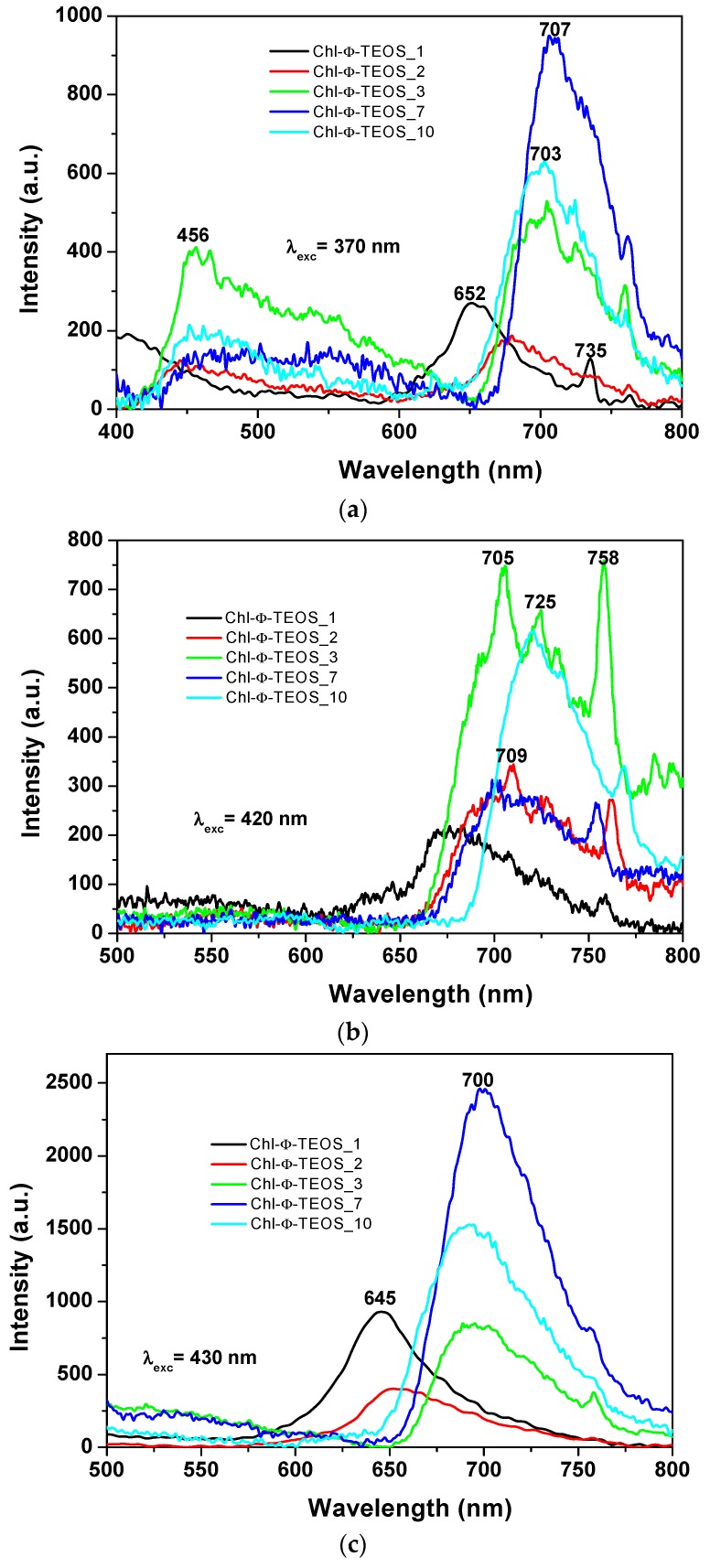
Emission spectra measured with an excitation light of: (**a**) 370 nm; (**b**) 420 nm; and (**c**) 430 nm, of samples synthesized using the Φ-TEOS alkoxide (Chl-Φ-X).

In the spectra of the xerogels modified with *phenyl* groups and containing different chlorophyll *a* amounts (i.e., Chl-Φ-X samples), while using an excitation light of 370 nm (Figure 12a), the same two regions of signals can be observed. A first one from 400 to 600 nm that can be linked either to the emissions of the silica network alone or to its interaction with chlorophyll. A second region extending from 600 to 800 nm and that can be due to the fluorescence of chlorophyll. The maximum emission intensity of these samples was observed in two zones and apparently depends on the chlorophyll loading. In the case of samples synthesized with a low amount of chlorophyll, i.e., 1 or 2 *v*/*V_f_*%, (samples Chl-Φ-1 and Chl-Φ-2) peak maxima were observed at 652 and 680 nm. However, these signals appear at around 705 nm when the chlorophyll concentration is higher than 9.414 × 10^−4^ M (i.e., when employing 3%, 7%, and 10% *v*/*V_f_* of chlorophyll-F solution). This situation suggests that chlorophyll can be trapped not only as monomers, but also as dimers or aggregates.

When exciting chlorophyll containing samples with light of 420 nm in wavelength (Figure 12b), the fluorescence intensity results higher for the Chl-Φ-3 and Chl-Φ-10 samples (synthesized from 3% and 10% of chlorophyll-F solution) bonded to the silica network that has been modified with *phenyl* groups. Spectroscopic evidence shows that the presence of chlorophyll *a* is stable and its fluorescent response is not interfering with the respective contribution of the silica matrix; it is as if the pigment macromolecules were “free” of any interaction.

A difference arises for samples functionalized with *allyl* groups, since the emission of samples containing *phenyl* groups appears as a broad band extending from 650 nm to 780 nm. There is a shift of the emission band to the infrared region; this depending of the organic group attached to the network. In both cases (*allyl* or *phenyl*), the broadening of the emission band is primarily due to the chlorophyll amount; at lower concentrations of the pigment, the band becomes narrower and well defined. The spectra of Ally-TEOS specimens are curiously defined over the near infrared region; in turn, the spectra of Φ-TEOS samples are defined toward the red region of the spectrum. Additionally, several other peaks attributed to chlorophyll appear at 705 nm, 709 nm, 725 nm, and 758 nm.

In all cases, a more regular signal pathway, although of different peak intensities, was observed when the samples synthesized with Φ-TEOS, were excited with 430 nm wavelength light (Figure 12c). Similarly, two other spectra pathways can be distinguished: those corresponding to samples having a low amount of chlorophyll, whose principal bands appear at around 646 to 660 nm and those observed for samples containing more than 9.414 × 10^−4^ M of the pigment (samples Chl-OSA-2) that show bands shifted to the red region, together with maximum intensity peaks at around 690–700 nm. The band pathways obtained suggest that these signals can be associated to the same emitting species, but arranged in a different way, i.e. as chlorophyll monomers or dimers. This result is more evident at low concentrations of chlorophyll, since a more regular signal pathway can be observed for samples synthesized from as low as 2% *v*/*V_f_* of chlorophyll solution. Even though a little amount of pyridine was added to each sample in order to inhibit the protonation and demetallation of chlorophyll and consequent phephorphide or pheophytin formation, this possibility could be the cause of the red shift of the fluorescence spectrum and requires deeper analysis. In all cases, the observed differences apparently seem to be a function of the chlorophyll concentration.

**Figure 13:**
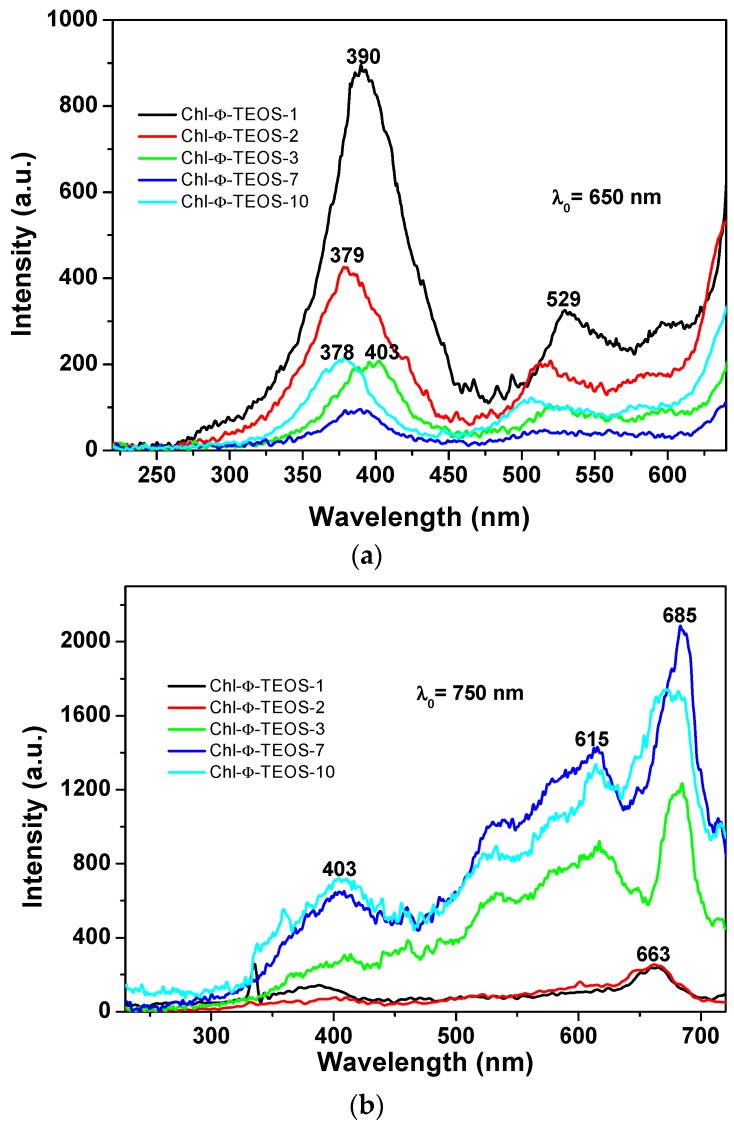
Excitation spectra obtained by using light of wavelength of: (**a**) 650; or (**b**) 750 nm, on silica samples modified on their surface with *phenyl* groups and containing diverse chlorophyll *a* concentrations covalently bonded to the pore walls of the network (Chl-Φ-X).

The events described above were similar to those observed in silica samples modified with *phenyl* groups, but when employing an excitation light of λ_exc_ = 650 nm or 750 nm (Figure 13a,b), in which the higher intensities were observed for those samples synthesized when using 1% an 2% *v*/*V_f_* of the chlorophyll-F solution (samples Chl-Φ-1 and Chl-Φ-2). In each excitation spectrum obtained with light of 650 nm, a principal signal localized at around 378 to 400 nm that corresponds to the Soret band as well as two bands at around 529 and 600 nm, which can be associated to the Q bands observed in the absorption spectra, can be seen. The spectrum of the sample synthesized with 3% *v*/*V_f_* of chlorophyll-F (sample Chl-Φ-3) appears slightly shifted into the red region while the Soret band is seen at 403 nm. However, when the spectra were obtained by using light of 750 nm, the most intense band appears in the red region of the spectrum and localized between 615 and 685 nm, with a smaller band at around 403 nm. Nonetheless, there exists only a tiny difference in intensity for the bands localized at around 390 nm and 663 nm for the samples synthesized from low amounts of chlorophyll (Chl-Φ-1 and Chl-Φ-2). Thus, at higher chlorophyll concentrations, the emission bands appear of higher intensity and shifted to the near infrared region, something that could be useful for other applications of these hybrid materials, as for instance, in optoelectronic or solar cell devices.

### 2.4. Textural Characterization by N_2_ Sorption

**Figure 14:**
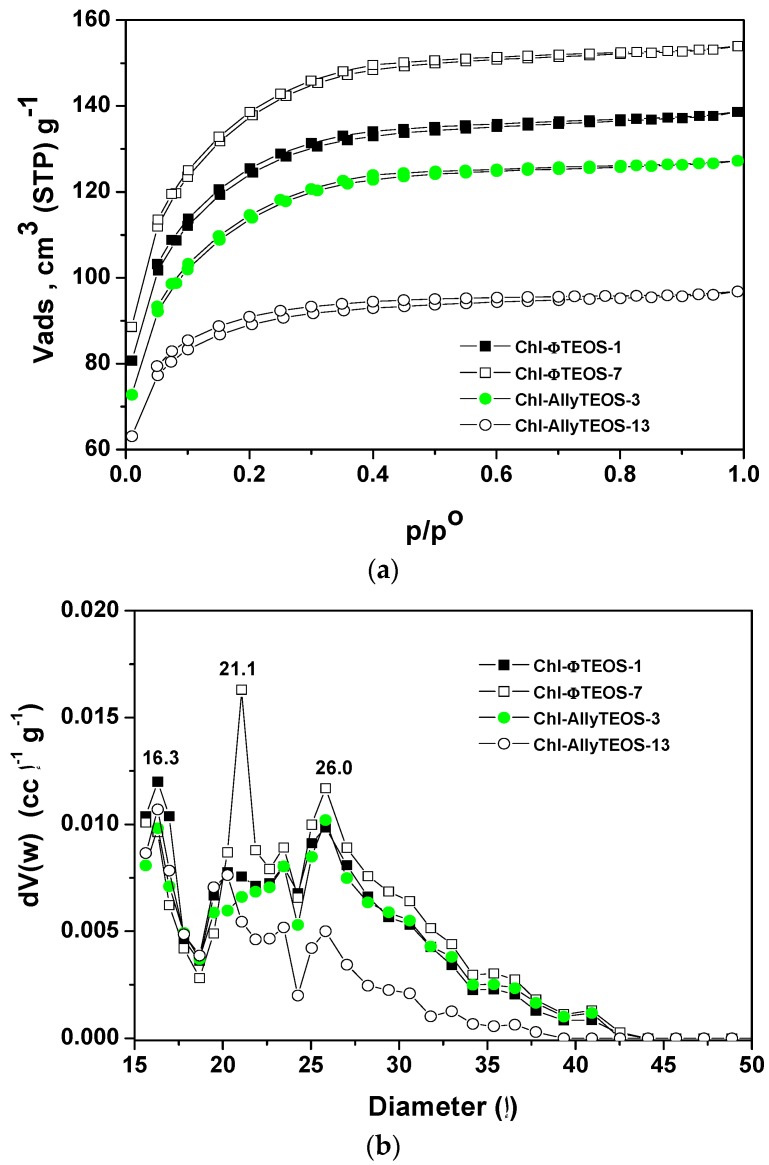
Sorption–desorption isotherms at 76 K (**a**) and pore size distributions (**b**) of silica networks modified on the surface with *allyl* (Chl-Ally-X) or *phenyl* (Chl-Φ-X) groups and with either a high or a low concentration of the chlorophyll *a* moiety covalently bonded.

The N_2_ sorption-desorption isotherms measured at 76 K on the organo-modified silica network containing chlorophyll at different concentrations (Figure 14a) depicted similar sorption curves, with different N_2_ total sorption capacities. The isotherms can be classified as IUPAC Type I with a very narrow hysteresis cycle [44,45], which, many times, are representative of materials including trapped macrocyclic species.

In the case of silica samples modified with attached *allyl* groups, the increase of chlorophyll concentration produces a decay of the sorption capacity of the network, as chlorophyll interferes with the penetration of N_2_ into the silica pores. Contrastingly, for the silica substrates modified with *phenyl* groups an opposite event occurs; the N_2_ sorption capacity of the network increases with the increase of the chlorophyll concentration.

The respective average pore size distributions (Figure 14b), calculated from the Non-Local Density Functional Theory (NLDFT) approach applied to the boundary desorption curve while assuming spherical voids, indicate mode pore sizes of about 1.6, 2.1, and 2.6 nm. In the case of silica samples modified with *allyl* groups and low chlorophyll loading (Chl-Ally-3), two pore widths are predominant: those of 1.6 and 2.6 nm, However, the pore sizes were the smallest for the network synthesized from high chlorophyll *a* loading (Chl-Ally-13). As postulated above, apparently, a higher amount of chlorophyll interferes with the access of adsorbate to the cavities, as several molecules of the macrocyclic species remain trapped in each pore. This event is only possible if the *allyl* groups existing in the gellifying mixture and finally being attached to the final network facilitate the aggregation of chlorophyll molecules, which is only possible if an intense interaction between chlorophyll and *allyl* groups exists. The pore size distribution of silica samples modified with *phenyl* groups confirm that an increase of chlorophyll loading induces an increase of the population of pores of sizes between 2.1 and 2.6 nm (Chl-Φ-1 to Chl-Φ-7). This situation points out that pores of these sizes were created around chlorophyll molecules; however, this fact is a consequence of the interaction of chlorophyll molecules with *phenyl* groups of the gellifying mixture during the consolidation process leading to the final xerogel network. Furthermore, apparently, the presence of these *phenyls* facilitates the solvation of chlorophyll, while signifying that only one molecule can remain trapped inside each pore.

### 2.5. Hypothetical Situation of the Chlorophyll inside the Pores

**Figure 15:**
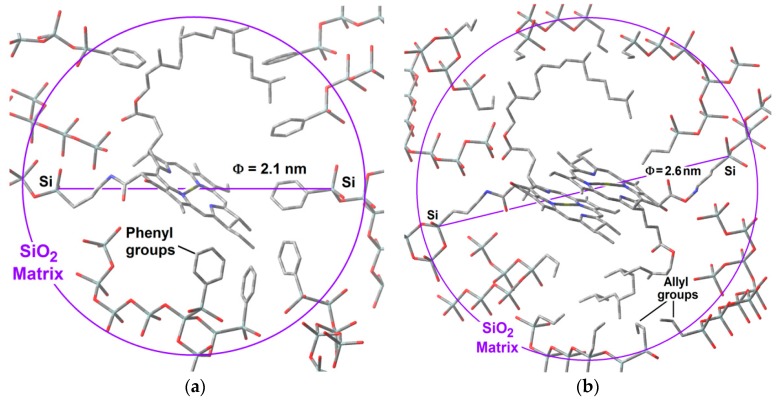
Hypothetical situation, modeled through *Gauss View* of monomers of chlorophyll *a* covalently bonded to the silica matrix modified with *phenyl* groups (**b**) or chlorophyll dimers bonded to the network modified with *allyl* groups. In both cases the distance between the APTES and the most opposite silicon atoms were of 2.10 and 2.65 nm, respectively.

With all of the above results, it is possible to advance a model of the hypothetical state of chlorophyll *a* inside the silica pores. The displayed monomeric structure of chlorophyll *a* has a size of around 3.2 nm and remains in this configuration all along the initial steps of the sol-gel process; during the shrinkage step, the collapse of pore cavities induces that the chlorophyll phytol axis (see Figure 1) to be folded. With this idea in mind and using simple *GaussView* 5.0 software (Free download software, Gaussian Inc., Wallingford, CT, USA), we have modeled the cavities created around monomeric and dimeric chlorophyll *a* structures (Figure 15a,b). For those networks containing either chlorophyll monomers modified on the surface with *phenyl* groups, or chlorophyll dimers surface functionalized with *allyl* groups, the distances from the silicon atom that allow the bonding of the macrocyclic structure to the silica network via one of the Si atoms located at the opposite side corresponded to 2.10 and 2.57 nm, respectively. These sizes coincided with the two principal mode pore diameters calculated from the N_2_ adsorption pore size distribution. This fact could explain the effects observed with respect to the adsorption capacity of each network as well as the characteristics of the fluorescence spectra.

Furthermore, these hypothetical models suggest that the close localization of *allyl* groups could be responsible of the diminishment of the N_2_ sorption capacity of this kind of systems when the chlorophyll *a* concentration increases. If this supposition is true, it means that in the most concentrated networks, the dimers of chlorophyll *a* could be located very near to each other inside neighboring cavities. This is similar in the systems synthesized using Φ−TEOS, but, in these systems, the size of the *phenyl* groups apparently remains far from the structure of the chlorophyll but placed at distances that allow its monomeric solvation. In this way, the situation of chlorophyll *a* in these systems resulted more adequate for the efficient display of its fluorescence. In all cases, these results shows that it is possible the design of the satisfactory environment to trap monomers or dimers of this transcendental natural molecule, with consequent physicochemical properties and technological applications.

## 3. Experimental Section

### 3.1. Reactives

3-aminopropyl-triethoxysilane (APTES), tetraethoxysilane (TEOS), and organo-substituted silicon alkoxides (OSA), such as *allyl*-trimethoxysilane (Ally-TEOS) and *phenyl*-triethoxysilane (Φ-TEOS), as well as all solid reagents employed in this work, were obtained from Aldrich, while all solvents were purchased from Fluka.

### 3.2. Chlorophyll *a* Extraction

The extraction and purification of chlorophyll *a* was made as described previously [28]; it started from common grass (*Poa vulgaris*) dispersed in ethanol and the corresponding extract was concentrated to a volume of about 200 mg/mL. Both UV absorption and fluorescence spectra of this chlorophyll extract were measured and taken as references.

### 3.3. Chlorophyll Covalent Bonding to Organo-Modified Silica Xerogels.

As previously demonstrated by our research group, chlorophyll *a* can be trapped inside the pores, while being bonded to the surface of monolithic, translucent organo-modified silica xerogels synthesized from tetraethoxysilane (TEOS) and organo-substituted silica alkoxides [25,27,28]. These last OSA compounds include, for instance, *allyl*-trimethoxysilane (Ally-TEOS) or *phenyl*-triethoxysilane (Φ-TEOS) [27,28]. To perform this functionalizing process, initially, a precursory (chlorophyll-F) species was first created by mixing 50 mL of an ethanolic solution of 0.230 g of chlorophyll and 0.061 mL of APTES; the reacting system was then taken to reflux conditions under magnetic stirring at a temperature of about 70 °C for 24 h; the reaction was monitored through FTIR spectroscopy. In a second step, the desired monolithic and translucent organo-modified silica xerogels were prepared from mixing different volumes of the chlorophyll-F ethanol solution with the next molar ratio composition: [H_2_O:TEOS + OSA: HCl: chlorophyll-F] = [19.6: 1:10^−3^: 10^−3^ to 10^−6^], which rendered a final total volume V_f_ (shown in Table 1). As previously demonstrated, in order to get translucent xerogels, the OSA volume of *allyl*- or *phenyl*-triethoxysilane (i.e., Ally-TEOS or Φ-TEOS) should be adjusted to reach no more than 1.0% *v*/*V_f_* [26,27,28]; importantly, 0.063 mL of pyridine (*py*) was added to inhibit the possible demetallation of chlorophyll. In order to improve the preparation method, the volume of the initial chlorophyll solution (5.135 × 10^−3^ mol/L) was varied from 1 to 15 *v*/*V_f_*% with respect to the total mixture volume. To identify each sample, the organic group attached to the network (The Ally- or Φ-groups coming from the OSA alkoxide used) was specified and the number (X or 1 to 13) accompanying each label (Chl-Ally or Φ-X) specifies the percentage of chlorophyll *a* solution added to the gellifying mixture, as shown in Table 1.

Every one of the mixtures listed in Table 1 was prepared thrice and kept inside 4 mL plastic cells and covered with *Parafilm* membrane and maintained under dark conditions. The process was monitored by UV absorption and, perforations were made through the *Parafilm*, until the emerging gel separated from the cell walls. The xerogel sample remained in the cell under this condition until no remaining solvent was left. As soon as the gel contraction ended, the ensuing substrates were dried for three weeks at room temperature, followed by three days at 70 °C, and finally by one day at 125 °C. The incumbent physicochemical properties of the resultant specimens were determined through UV, NIR, and fluorescence spectroscopies as well as by N_2_ sorption.

### 3.4. Characterization

All absorbance spectra characterization was carried out in a Cary-Varian 500E spectrophotometer in the range of 200 to 800 nm (UV-Vis range) and from 1000 nm to 2500 nm (i.e., 100,000 to 4000 cm^−1^) (NIR range). The Fourier transform infrared (FTIR) spectra were obtained from a Perkin-Elmer GX FTIR spectrometer.

Continuous fluorescence spectroscopy studies of the solid xerogel samples were performed at room temperature in a model 650-10S Perkin-Elmer spectrofluorometer, in the 400 to 800 nm Vis range. Photoluminescence measurements were also carried out through the corresponding excitation spectra. The light source employed was a 10 W pulsed Xenon lamp having a width at half peak intensity of 0.01 ms.

N_2_ adsorption-desorption experiments were measured in an Automatic Volumetric Quantachrome Autopore 1L-C instrument at 76 K (boiling point of N_2_ at México City’s 2250 m altitude). The average pore diameters of the silica matrix with the macrocyclic species bonded to the pore network were calculated from the NLDFT approach applied to the N_2_ desorption boundary branch of the isotherms [44,45] while assuming spherical pore cavities.

The High Resolution Scanning Electron Microscopy images (HRSEM) were obtained via a Jeol 7600F with an Oxford instruments X-MAX 20 mm^2^ (SDD) detector for allowing a high-resolution mapping. Electron Dispersion X-ray Scattering (EDX) measurements were employed to visualize the presence and distribution of organic groups and chlorophyll *a*, specially the existence of Magnesium and the presence of the elements forming part the SiO_2_ inorganic matrix. The elemental analysis data were normalized and results from the average of nine spectra taken over the surface of each sample; for simplicity, the element distribution, and its respective weight percent in each sample were omitted.

## 4. Conclusions

The results herein showed demonstrated that chlorophyll *a* was successfully introduced and bonded into the silica pore network, a substrate that was previously organically modified with *phenyl* or *allyl* groups.

A higher luminescence emission was observed in those systems containing a low chlorophyll concentration. At high chlorophyll concentrations, luminescence decreases possibly as a consequence of either the dimerization of these macrocyclic species or by interactions with remnant Si-OH groups localized on the pore surface.

In order to optimize the physicochemical properties that an active molecule such as chloriophyll *a* displays in solution, but instead inserted inside a solid substrate, an adequate amount of this species results to be an important parameter. Thus, it is important to delimit the maximum amount of chlorophyll to be incorporated inside a monolithic silica sample, since the chlorophyll dimerization can interfere with an efficient display of the optical properties of the resultant hybrid materials. In our experiments, this limiting condition corresponded to the samples synthesized from no more than 2% *v*/*V_f_* of chlorophyll-APTES solution, which accounted for 8.558 × 10^−5^ M in the initial gellifying mixture and to 9.414 × 10^−4^ M in the final monolithic xerogels.

Furthermore, as is well known, fluorescence, as well as other important physicochemical properties of chlorophyll *a* existing as dimers could still be very useful for different applications in fields such as solar cells or optoelectronic devices. The results herein demonstrate that the presence of *allyl* or *phenyl* groups attached to the silica pore surface allows the preferential trapping of chlorophyll *a* in the form of dimers and monomers, respectively.

The results depicted in this document show that a good control of the initial conditions of synthesis allows obtaining hybrid materials with adequate textural parameters for the sorption of diverse species, such as N_2_, water and, surely, oxygen and CO_2_. In this way, the methodology here presented could make it possible to better exploit the properties of these transcendental chemical species in novel hybrid materials suitable to be applied in strategic fields such as catalysis, optics, compound sensing, and medicine.

## Figures and Tables

**Table 1:** Gelling mixtures employed for the synthesis of translucent monolithic organo functionalized silica xerogels including chlorophyll *a* covalently bonded to the pore walls of SiO_2_ substrates.

Sample	HCl:TEOS (mL)	H_2_O (mL)	OSA * (mL)	Chlorophyll-F (mL)	*V_f_* ** (mL)	[Chlorophyll *a*]_i_ (mol/L)	[Chlorophyll *a*]_f_ (mol/L)
Blank	5	1	0.0	0.0	6.0	0.0	0.0
Chl-OSA-1	5	0.85	0.1	0.05	6.0	4.279 × 10^−5^	4.701 × 10^−4^
Chl-OSA-2	5	0.8	0.1	0.1	6.0	8.558 × 10^−5^	9.414 × 10^−4^
Chl-OSA-3	5	0.7	0.1	0.2	6.0	1.712 × 10^−4^	1.883 × 10^−3^
Chl-OSA-7	5	0.5	0.1	0.4	6.0	3.423 × 10^−4^	3.765 × 10^−3^
Chl-OSA-10	5	0.3	0.1	0.6	6.0	5.135 × 10^−4^	5.648 × 10^−3^
Chl-OSA-13	5	0.1	0.1	0.8	6.0	6.846 × 10^−4^	7.531 × 10^−3^
Chl-OSA-15	5	0.0	0.1	0.9	6.0	7.702 × 10^−4^	8.472 × 10^−3^

* OSA = Organo substituted alkoxides, i.e., Ally-TEOS or Φ-TEOS. The i and f subindexes make reference to the chlorophyll *a* concentration existing in the initial gellifying mixture as well as in the final monolithic xerogels. ** *V_f_* = Total mixture volume.

## References

[1] Milgrom R.L. (1997). The Colour of Life. An Introduction to the Chemistry of Porphyrins and Related Compounds.

[2] Bedioui F. (1995). Zeolite-encapsulated and clay-intercalated metal porphyrin, phthalocyanine and Schiff-base complexes as models for biomimetic oxidation catalysts: An overview. Coord. Chem. Rev..

[3] Dunbar A.D.F., Brittle S., Richardson T.H., Hutchinson J., Hunter C.A. (2010). Detection of volatile organic compounds using porphyrin derivatives. J. Phys. Chem. B.

[4] Wang X.J., Yates L.M., Knobbe E.T. (1994). Study of nonlinear absorption in metalloporphyrin-doped sol-gel materials. J. Lumin..

[5] Ethirajan M., Chen Y.Y, Joshi P., Pandey R.K. (2011). The role of porphyrin chemistry in tumor imaging and photodynamic therapy. Chem. Soc. Rev..

[6] Yoon I.I., Jia Z.L., Shim Y.K. (2013). Advance in photosensitizers and light delivery for photodynamic therapy. Clin. Endosc..

[7] Loewen G.M., Pandey R.K., Bellnier D., Henderson B.W., Dougherty T. (2006). Endobronchial photodynamic therapy for lung cancer. J. Lasers Surg. Med..

[8] Castano A.P., Mroz P., Hamblin M.R. (2006). Photodynamic therapy and anti-tumour immunity. Nat. Rev. Cancer.

[9] Harriman A. (1980). Luminescence of porphyrins and metalloporphyrins Part I. J. Chem. Soc. Faraday. Trans. 1.

[10] Lavi A., Weitman H., Holmes R.T., Smith K.M., Ehrenberg B. (2002). The depth of porphyrin in a membrane and the membrane’s physical properties affect the photosensitizing efficiency. Biophys. J..

[11] Marijnissen J.P.A., Star W.M. (1987). Quantitative light dosimetry in vitro and in vivo Lasers. Med. Sci..

[12] Kobayashi M., Akutsu S., Fujinuma D., Furukawa H., Komatsu H., Hotota Y., Kato Y., Kuroiwa Y., Watanabe T., Ohnishi-Kameyama M., Dubinsky Z. (2013). Pysicochemical Properties of Chlorophylls in Oxygenic Photosynthesis—Succession of Co-Factors from Anoxygenic to Oxygenic Photosynthesis; Agricultural and Biological Sciences. Photosynthesis.

[13] Calogero G., di Marco G., Caramori S., Cazzanti S., Argazzi R., Bignozzi C.A. (2009). Natural dye senstizers for photoelectrochemical cells. Energy. Environ. Sci..

[14] Shanmugam S., Xu J., Boyer C. (2015). Utilizing the electron transfer mechanism of chlorophyll *a* under light for controlled radical polymerization. Chem. Sci..

[15] Li W., Zhu G., Li J., Wang Z., Jin Y. (2016). An amidochlorin-based colorimetric fluorescent probe for selective Cu^2+^ detection. Molecules.

[16] Rebeiz C.A. (2014). Chlorophyll Biosynthesis and Technological Applications.

[17] Snyder E.G. (1942). Bacteriostatic Substance and Process for Making the Same. U.S. Patent.

[18] Saka I., Nakajima S. (1987). Pheophorbid Derivatives and Alkaline Salt Thereof. U.S. Patent.

[19] Bae S.M., Kim Y.W., Lee J.M., Namkoong S.E., Han S.J., Kim J.K., Lee C.H., Chun H.J., Jin H.S., Ahn W.S. (2004). Photodynamic effects of radachlorin^®^ on cervical cancer cells. Cancer Res. Treat..

[20] Liu T.W.B., Chen J., Burgess L., Cao W., Shi J., Wilson B.C., Zheng G. (2011). Multimodal bacteriochlorophyll theranostic agent. Theranostics.

[21] Uliana M.P., Pires L., Pratavieira S., Brocksom T.J., de Oliveira K.T., Bagnato V.S., Kurachi C. (2014). Photobiological characteristics of chlorophyll aderivatives as microbial PDT agents. Photochem. Photobiol. Sci..

[22] García-Sánchez M.A., Campero A. (2000). Aggregation properties of metallic tetrasulfophthalocyanines embedded in sol-gel silica. Polyhedron.

[23] García-Sánchez M.A., Campero A. (2001). Insertion of Lanthanide porphyrins in silica gel. J. Non Cryst. Solids.

[24] García-Sánchez M.A., de la Luz V., Coahuila-Hernández M.I., Rojas-González F., Tello-Solís S.R., Campero A. (2011). Effects of the structure of entrapped substituted porphyrins on the textural characteristics of silica network. J. Photochem. Photobiol. A Chem..

[25] Quiroz-Segoviano R.I.Y., Serratos I.N., Rojas-González F., Tello-Solís S.R., Sosa-Fonseca R., Medina-Juaréz O., Menchaca-Campos C., García-Sánchez M.A. (2014). On tunin fluorescence emission of porphyrins free bases bonded to the pore walls of organo-modified silica. Molecules.

[26] González-Santiago B., García-Sánchez M.A. (2011). Macrocycle-pore network interactions: Aluminum tetrasulfophthalocyanine in organically modified silica. J. Non-Cryst. Solids.

[27] Quiroz-Segoviano R.I.Y., García-Sánchez M.A., Rojas-González F. (2012). Cobalt porphyrin covalently bonded to organo modified silica xerogels. J. Non-Cryst. Solids.

[28] Serratos I.N., Rojas-González F., Sosa-Fonseca R., Esparza-Schulz J.M., Campos-Peña V., Tello-Solís S.R., García-Sánchez M.A. (2013). Fluorescence optimization of chlorophyll covalently bonded to mesoporous silica synthesized by the sol-gel method. J. Photochem. Photobiol. A Chem..

[29] Humphrey W., Dalke A., Schulten K. (1996). VMD: Visual molecular dynamics. J. Mol. Graph..

[30] Soret J.L. (1883). Analyse spectrale: Sur le spectre d’absorption du sang dans la partie violette et ultra-violette. Compt. Rend..

[31] Gouterman M. (1961). Spectra of porphyrins. J. Mol. Spectrosc..

[32] Gouterman M., Wagnière G.H., Snyder L.C. (1963). Spectra of porphyrins: Part II. Four orbital model. J. Mol. Spectrosc..

[33] Senge M.O., Ryan A.A., Letchford K.A., MacGowan S.A., Mielke T. (2014). Chlorophylls, symmetry, chirality, and photosynthesis. Symmetry.

[34] Smith K.M. (1976). Porphyrins and Metalloporphyrins.

[35] Dolphin D. (1979). The Porphyrins, Physical Chemistry, Parts A and B.

[36] Riischepov A.S., Gurinovich G.P. (1975). Aggregation of chlorophyll isomers in a mixture of polar solvents. Zh. Prikl. Spektrosk..

[37] Mass O., Pandithavidana D.R., Ptaszek M., Santiago K., Springer J.W., Jiao J., Tang Q., Kirmaier C., Bocian D.F., Holten D., Lindsey J.S. (2011). De novo synthesis and properties of analogues of the self-assembling chlorosomal bacteriochlorophylls. New J. Chem..

[38] Correia R.F., Viseu M.I., Andrade S.M. (2014). Aggregation/disaggregation of chlorophyll *a* in model phospholipid-detergent vesicles and micelles. Photochem. Photobiol. Sci..

[39] Peri J.B. (1966). Infrared Study of OH and NH_2_ Groups on the Surface of a Dry Silica Aerogel. J Phys. Chem..

[40] Orgaz F., Rawson H. (1986). Characterization of various stages of the sol-gel process. J. Non-Cryst. Solids.

[41] Wood D.L., Rabinovich E.M. (1986). Infrared studies of alkoxide gels. J. Non-Cryst. Solids.

[42] Salas-Bañales E., Quiroz-Segoviano R.I.Y., Díaz-Alejo L.A., Rojas-González F., Estrella-González A., Campero A., García-Sánchez M.A. (2015). Comparative Study of the Optical and Textural Properties of Tetrapyrrole Macrocycles Trapped Within ZrO_2_, TiO_2_, and SiO_2_ Translucent Xerogels. Molecules.

[43] Katz J.J., Shipman L.L., Cotton T.M., Janson T.J. (1978). The Porphyrins.

[44] Sing K.S.W., Everett D.H., Haul R.A.W., Moscow L., Pierotti R.A., Rouquerol J., Siemieniewska T. (1985). Reporting physisorption data for gas/solid systems with special reference to the determination of surface area and porosity. Pure Appl. Chem..

[45] Gregg S.J., Sing K.S.W. (1967). Adsorption, Surface Area and Porosity.

